# Flame-retardant MXene/polyimide film with outstanding thermal and mechanical properties based on the secondary orientation strategy†

**DOI:** 10.1039/d1na00415h

**Published:** 2021-08-10

**Authors:** Yue Zhu, Xingbin Zhao, Qingyu Peng, Haowen Zheng, Fuhua Xue, Pengyang Li, Zhonghai Xu, Xiaodong He

**Affiliations:** National Key Laboratory of Science and Technology on Advanced Composites in Special Environments, Center for Composite Materials and Structures, Harbin Institute of Technology Harbin 150080 P. R. China pengqingyu@hit.edu.cn; Shenzhen STRONG Advanced Materials Research Institute Co., Ltd. Shenzhen 518000 P. R. China

## Abstract

With the development of multifunction and miniaturization in modern electronics, polymeric films with strong mechanical performance and high thermal conductivity are urgently needed. Two-dimensional transition metal carbides and nitrides (MXenes) have attracted extensive attention due to their tunable surface chemistry, layered structure and charming properties. However, there are few studies on using MXenes as fillers to enhance polymer properties. In this paper, we fabricate a three-dimensional foam by the freeze-drying method to enhance the interfacial interaction between adjacent MXene sheets and polyimide (PI) macromolecules, and then a composite film with a dense and well-ordered layer-by-layer structure is produced by the hot-pressing process. Based on the secondary orientation strategy, the resultant MXene/PI film exhibits an enhanced thermal conductivity of 5.12 ± 0.37 W m^−1^ K^−1^ and tensile strength of 102 ± 3 MPa. Moreover, the composite film has good flexibility and flame retardancy owing to the synergistic effect of MXene sheets and PI chains. Hence, the MXene/PI composite film with the properties of flexibility, flame-retardancy, high mechanical strength and efficient heat transmission is expected to be used as the next thermal management material in a variety of applications.

## Abstract 2

A three-dimensional foam is fabricated to enhance the interfacial interaction between adjacent MXene sheets and polyimide (PI) macromolecules; then a composite film with a dense and well-ordered layer-by-layer structure, good flexibility and flame retardancy is produced.

## Graphical abstract

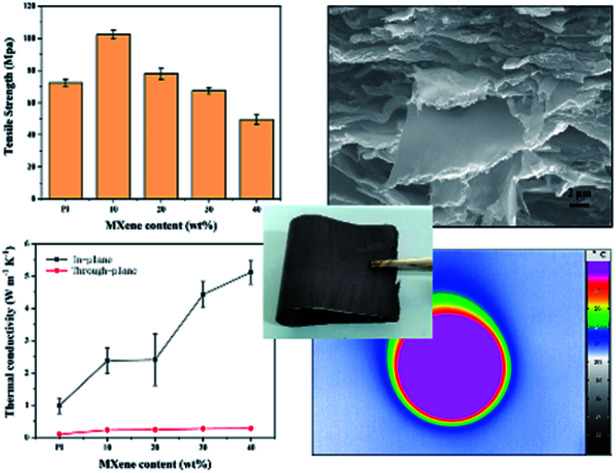

## 1. Introduction

With the advent of the 5G era, modern electronics are developing rapidly toward the multifunction and miniaturization direction.^1–3^ The technical integration of high-power chips, wireless charging, bluetooth and multi-angle folding has caused a severe challenge to the heat dissipation and strength of electronic devices.^4,5^ Hence, to effectively dissipate heat in time and transform the brittleness of materials, composites synchronously with excellent thermal and mechanical properties are urgently needed. Compared with the other three material systems of wood, metal and silicate, polymeric materials are attracting more and more attention due to their light weight, flexibility, easy processing and excellent corrosion resistance.^6^ However, most polymers are inherently poor conductors of heat and electrons, which limits their applications in electronic devices greatly. In addition, these electronic devices also face serious fire hazards caused by accidental electrical leakage or aging generally.^7,8^ However, it is difficult to restrain the fire spread in most polymeric materials once it occurs. Therefore, research and preparation of multifunctional materials with high strength, good thermal conductivity and flame retardancy at the same time have far-reaching academic significance and wide practical value.

Polyimide (PI) as one of the most popular functional materials has been widely applied in the fields of aerospace, optics and microelectronics, due to its excellent properties, such as chemical resistance, thermal stability and mechanical performance.^9^ Recently, incorporating fillers of carbonaceous materials (such as carbonaceous carbon fibers, graphene and carbon nanotubes), metals (such as Cu nanowires) and insulating thermal materials (such as boron nitride) into a polymer matrix has been considered as one of the most effective and feasible methods to improve the thermal conductivity of composites.^10–16^ For instance, Wei *et al.* obtained a reduced graphene oxide/PI (rGO/PI) film with a thermal conductivity of 2.78 W m^−1^ K^−1^ when the content of rGO was 8 wt%.^12^ Wang *et al.* obtained an anisotropic thermal conductivity of 2.81 W m^−1^ K^−1^ in PI composites with 30 wt% boron nitrides.^13^ In 2020, He *et al.* reported a highly thermally conductive (11.203 W m^−1^ K^−1^) PI composite film with graphene oxide (GO) nanosheets and boron nitride (BN) platelets as binary fillers.^15^ However, in general, carbon fillers are incompatible with the polymer matrix, leading to serious aggregation of carbon fillers in composites, which increases the interfacial thermal resistance between fillers and the matrix and greatly limits the heat transfer performance of composites. Hence, it is still a big problem to achieve a perfect balance between the interfacial compatibility and intrinsic thermal conductivity of fillers.

Recently, MXenes, a class of 2D early transition metal carbide and nitride materials, have been discovered, and are fabricated by selectively etching “A” layers from layered ceramics called MAX phases.^17^ And the general formula of MAX phases is M_*n*+1_X_*n*_, where M represents the early transition metal, X represents carbon or nitrogen and *n* = 1, 2 or 3. Due to the unique layered atomic structure and adjustable surface functional groups (hydroxyl, oxygen or fluorine), MXenes show fascinating properties in many aspects, such as energy storage, electromagnetic interference shielding, adsorption performance, catalytic performance and thermal conductivity.^17–21^ In 2016, Zha *et al.* investigated the theoretical thermal conductivity (472 W m^−1^ K^−1^) of Sc_2_CT_2_ (T = F, OH) MXene using first-principles calculations,^21^ which provided a new direction for the preparation of thermal management materials.^17,22–27^ For instance, Song *et al.* fabricated a cellulose nanofiber (CNF)/Ti_3_C_2_ composite film using a vacuum-assisted filtration method, which exhibited a high thermal conductivity of 11.57 W m^−1^ K^−1^.^17^ Jin *et al.* reported multilayered poly(vinyl alcohol)/MXene films with a thermal conductivity of 4.57 W m^−1^ K^−1^.^22^ However, there is still a lack of in-depth exploration on using MXenes as fillers to enhance the thermal conductivity of the polymer matrix, especially in the aspects of high strength and heat transfer. Hence, in our work, because MXenes are synthesized in fluoride-containing aqueous solutions, MXenes exhibit good dispersibility in poly(amic acid) (PAA) solution, which can effectively improve the interfacial compatibility between fillers and the matrix and improve the thermal conductivity of composite materials.

Herein, the MXene/PI film with a dense and well-ordered layer-by-layer structure was fabricated by the interfacial interaction between adjacent MXene sheets and PI macromolecules and the secondary orientation strategy including the freeze-drying method and hot-pressing process. The stacked MXene interlocking structure not only increases the in-plane heat transfer path and further reduces the interfacial thermal resistance, but also enhances the friction between sheets to improve the fracture strength of MXene/PI films. The resultant MXene/PI film exhibits an enhanced thermal conductivity of 5.12 ± 0.37 W m^−1^ K^−1^ and tensile strength of 102 ± 3 MPa. More importantly, the composite film has good flexibility and flame retardancy due to the synergistic effect between MXene sheets and PI chains. Hence, this study provides a feasible and effective scheme for preparing flexible, flame-retardant polymeric films with excellent thermal and mechanical properties.

## 2. Results and discussion

### 2.1. Preparation process of the MXene/PI film

**Fig. 1:**
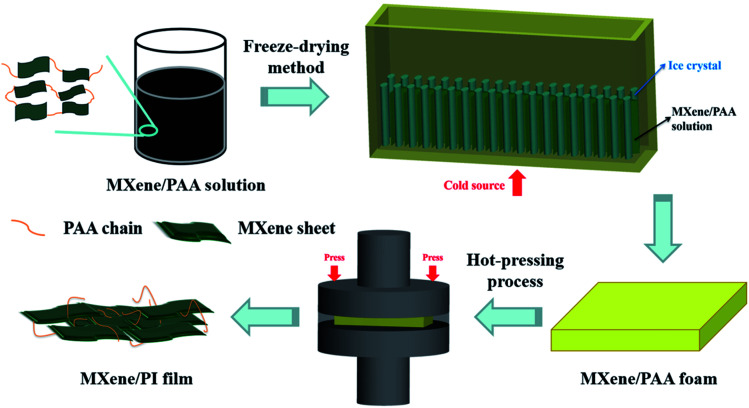
Schematic illustration of the preparation process of the MXene/PI film.

The MXene/PI film was fabricated by means of two steps of the freeze-drying method and hot-pressing process, that is, the secondary orientation strategy. The process of preparing the MXene/PI film is shown in Fig. 1. Firstly, the poly(amic acid) (PAA) solution is mixed with the MXene (Ti_3_C_2_T_*x*_) suspension to enhance the interfaces between MXene sheets. As described in the section of Materials and methods, the samples with different MXene contents are denoted as MXene-*x*/PAA, where *x* was 10, 20, 30, and 40, respectively. Subsequently, the homogeneous mixture is further frozen (cold source at the bottom) and lyophilized in a freeze-dryer. The anisotropic MXene/PAA foam is obtained due to the rearrangement effect of freeze casting.^20^ Finally, the MXene/PAA foam is subjected to the hot-pressing process in a (300 °C and 25 MPa) vacuum furnace to induce the polymerization of PAA to form PI macromolecules. The thickness of the film can be well controlled to several tens of micrometers by adjusting the pressure of the vacuum furnace. Thus, flame-retardant MXene/PI films with outstanding thermal and mechanical properties are obtained. And the experimental details can be found in the part of Materials and methods.

### 2.2. Morphology of MXene/PAA foam and MXene/PI films

**Fig. 2:**
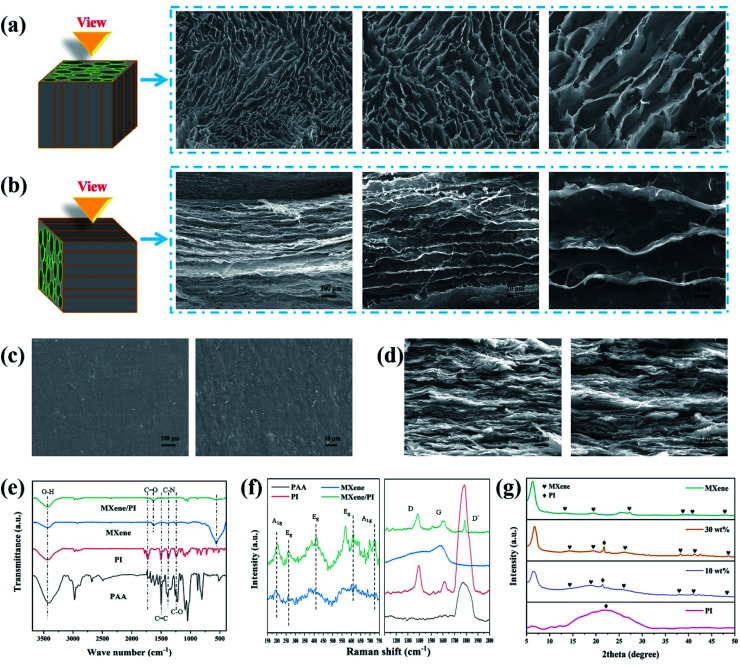
Morphology and structural characterization of MXene, PI, PAA, MXene/PAA foam and the MXene/PI film. SEM images of (a) vertical and (b) parallel to the direction for the MXene sheets of MXene-10/PAA foam at low and high magnification. SEM images of the (c) surface morphology and (d) cross-sectional images of the MXene-10/PI film at low and high magnification. (e) FTIR spectra and (f) Raman spectra of PAA, PI, MXene and MXene/PI. (g) XRD patterns of PI, MXene-10/PI, MXene-30/PI and MXene.

To further investigate the secondary orientation effect of the MXene/PI film, the morphology characterization of the MXene/PAA foam and MXene/PI film is performed with scanning electron microscopy (SEM). Owing to the secondary orientation process, the MXene/PI film exhibits a compact directional structure. On the one hand, the anisotropic MXene/PAA foam is fabricated by freeze-drying. Owing to the rearrangement effect of freeze casting,^28^ the MXene/PAA foam shows a porous structure with smooth cell walls (Fig. 2(a)) and a highly oriented structure similar to the tube bundles (Fig. 2(b)), different from the MXene foam with a loosely disordered porous structure with weakly interconnected sheets.^29^ On the other hand, to further control and improve the MXene sheet distribution and interface bonding of the MXene/PI film, MXene/PAA foam is subjected to the hot-pressing process and PAA is amidated into PI at the same time. The fabricated MXene/PI film exhibits a dense and well-ordered layer-by-layer structure without interlayer delamination (Fig. 2(d)) and a smooth and flat surface (Fig. 2(c)). Some particulate impurities can be observed in the high magnification SEM image in Fig. 2(c), which may be carbon particles on the graphite mold.

### 2.3. Structural characterization of MXene, PI, PAA and MXene/PI films

The chemical structure and possible interactions of the composite are further confirmed by Fourier transform infrared (FTIR) spectroscopy, Raman spectroscopy and X-ray diffraction (XRD). As shown in Fig. 2(e), the synthesized MXene contains 3433 cm^−1^, 1624 cm^−1^ and 561 cm^−1^ peaks, indicating –OH, –COOH and Ti–O functional groups, respectively.^30^ As for the PI film, the characteristic peaks at 1726 cm^−1^ and 1780 cm^−1^ are assigned to the imide C=O symmetric and asymmetric stretches, and two peaks at 1502 cm^−1^ and 1379 cm^−1^ can be observed due to the stretching vibration of C=C and C–N.^31,32^ After heat imidization treatment, the successful synthesis of the MXene/PI film is distinctly confirmed by the disappearance of the carboxylic acid and amine groups in PAA foam at 2500–3500 cm^−1^ and the presence of the characteristic peaks at 1639 cm^−1^ (C=O), 1379 cm^−1^ (C=C) and 1226 cm^−1^ (C–N).^33^ The reason for the shift of peaks in the MXene/PI film is that strong hydrogen bonds are formed by the functional groups of Ti_3_C_2_T_*x*_ sheets with the carbonyl groups of PAA chains.^29,31^ As shown in Fig. 2(f), for the range from 150 cm^−1^ to 750 cm^−1^, the MXene/PI film and MXene exhibit similar characteristic peaks of the A_1g_ (205 cm^−1^, 715 cm^−1^) band and E_g_ (270 cm^−1^, 407 cm^−1^, 620 cm^−1^) band.^34–36^ Simultaneously, the characteristic Raman peaks of the MXene/PI film at 1380 cm^−1^, 1594 cm^−1^ and 1787 cm^−1^ can also be observed, which is in agreement with the band of PI according to the literature.^31^ Besides, the stretching of sp^3^ carbon leads to a peak of MXene at 1582 cm^−1^.^37^ The structure of the MXene/PI film is monitored with the XRD pattern, as shown in Fig. 2(g). The diffraction peak around 21.4° is observed in the MXene/PI film, which corresponds to the amorphous structure of the PI film.^38^ Compared to the MXene, the (002) peak of the MXene/PI film shifts from 6.32° to 6.7°, corresponding to the reduced interlayer spacing from 1.4 to 1.32 nm.^34^ This result indicates that the arrangement of MXene sheets in the MXene/PI film is denser along the in-plane direction, corresponding to the SEM result, which means the fabrication of a compact and well-ordered layer-by-layer structure. Therefore, in this work, the fabricated MXene/PI film is expected to promote thermal and mechanical properties, owing to the formation of the heat transfer path and strong interaction between MXene sheets and PI chains.

### 2.4. Mechanical properties of MXene/PI films

**Fig. 3:**
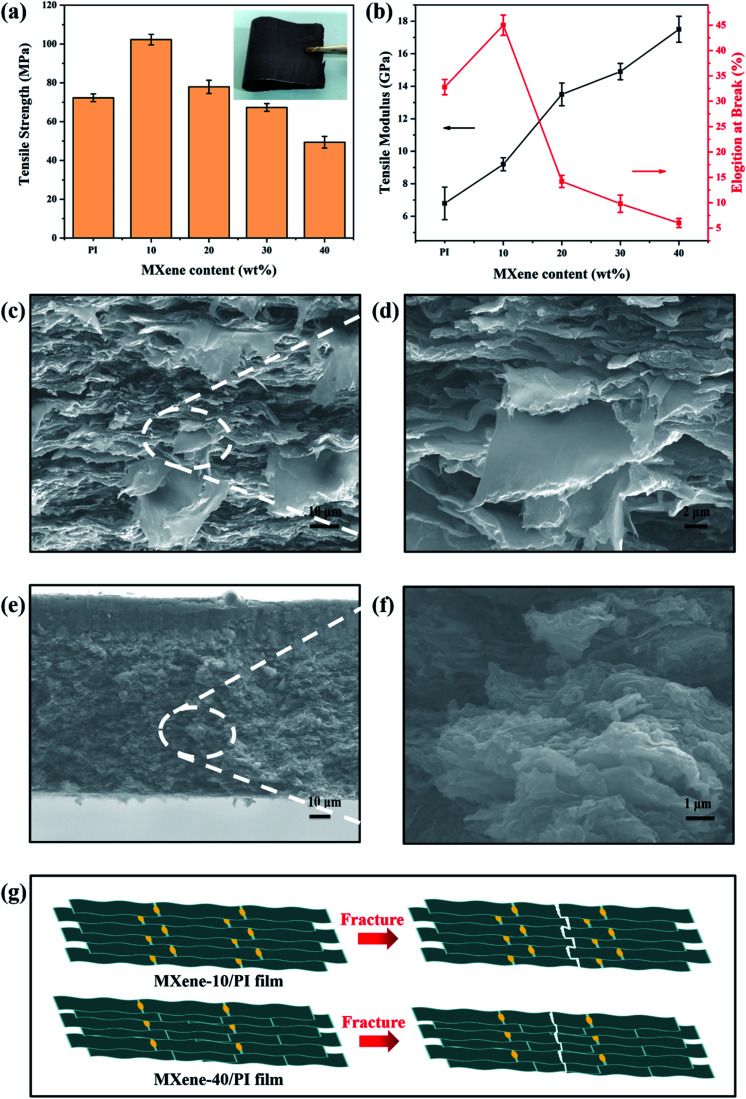
Mechanical properties of MXene/PI films. (a) Tensile strength, where the inset shows the digital image of the flexible MXene-10/PI film, and (b) tensile modulus and elongation at break of PI films and MXene/PI films with different MXene weight ratios. SEM images of the fractured surface of MXene-10/PI films at (c) low and (d) high magnification, respectively. SEM images of the fractured surface of MXene-40/PI films at (e) low and (f) high magnification, respectively. (g) Schematic illustration of the fracture mechanism of the MXene-10/PI film and Mxene-40/PI film.

**Fig. 4:**
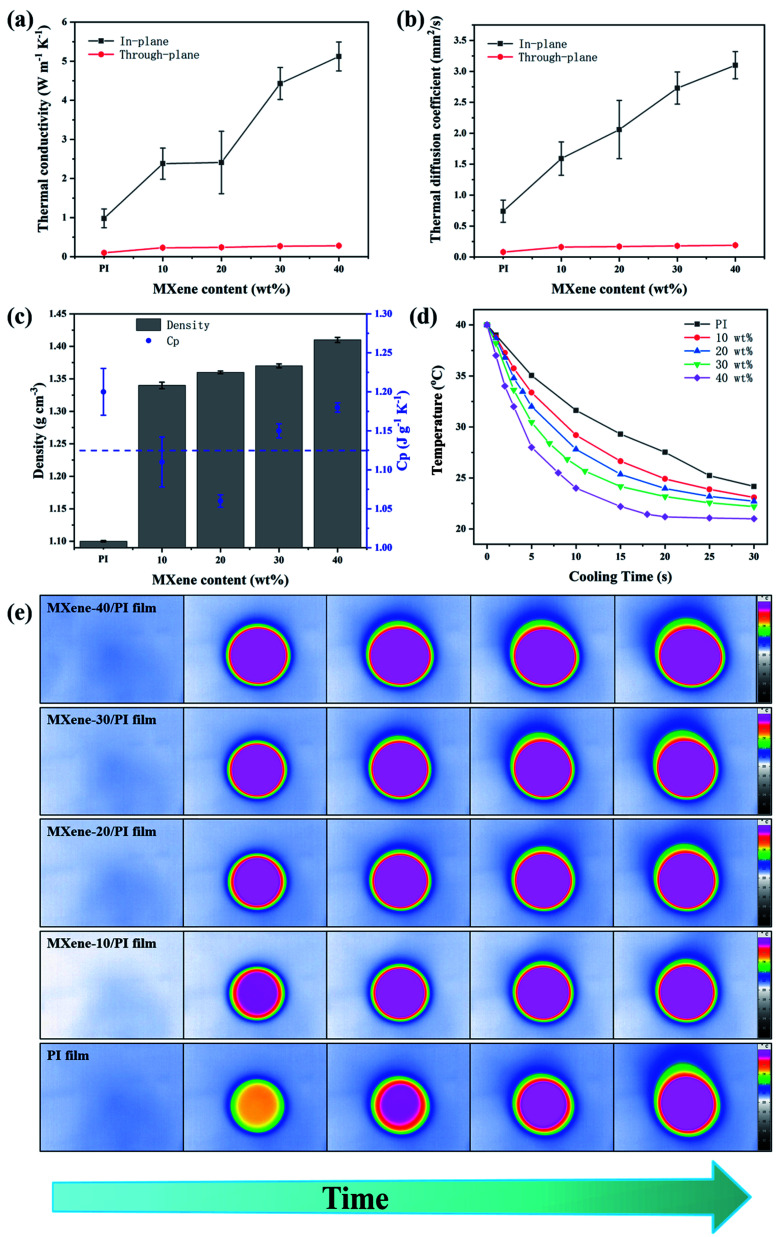
Thermal management performance of MXene/PI films. In-plane and through-plane (a) thermal conductivities and (b) thermal diffusion coefficient of PI films and MXene/PI films. (c) Densities and specific heat capacity *versus* MXene/PI films with different contents of MXene. (d) Variation in the temperature of the mesosphere of MXene/PI films with different weight ratios of MXene upon cooling when the center temperature is 39.8 °C. (e) Comparison of the infrared images of MXene-40/PI, MXene-30/PI, MXene-20/PI, MXene-10/PI and PI films upon heating. The center temperatures of all films were at the same level.

To investigate the reinforcing effect of MXene sheets and PI chains in the composite films, the tensile strengths, tensile modulus and elongation at break of the MXene/PI film with different MXene contents (10, 20, 30 and 40 wt%) were compared. Apparently, the MXene/PI film shows enhanced mechanical properties. As shown in Fig. 3(a), the tensile strength of the MXene-10/PI film (102 ± 3 MPa) is stronger than that of the PI film (73 ± 2 MPa), because of the synergistic effect of MXene sheets and PI chains. The typical stress–strain curves of PI films and MXene/PI films are shown in Fig. S1 of ESI.† However, the tensile strength gradually decreases with increasing MXene fraction. These results possibly originate from the fact that the excessive MXene would prevent the PI chains from packing tightly, thereby increasing the free volume, reducing the interaction between PI chains and increasing the brittleness of the MXene/PI film.^39^ Simultaneously, as shown in Fig. 3(b), the elongation at break of the MXene-10/PI film reaches 45 ± 2%, while the excessive MXene makes PI chains more rigid, resulting in increased brittleness of the film.^39^ In addition, the tensile modulus of the MXene/PI film gradually increases due to the fact that the density increases with the MXene content (Fig. 4(c)),^40^ and thus the MXene-40/PI film exhibits the highest modulus of 17.5 ± 0.8 GPa.

To further demonstrate the synergistic effect of MXene sheets and PI chains in the composite films, the tensile fracture morphologies were characterized by SEM. As shown in Fig. 3(c and d), a large number of pull out flaky structures can be observed in the fracture surface of the MXene-10/PI film, while the fracture of the MXene-40/PI film is brittle like and shows a small and flat pull out structure (Fig. 3(e and f)), indicating that the MXene/PI film can absorb the breaking energy to enhance the tensile strength during the tensile process.^41,42^Fig. 3(g) demonstrates the possible fracture model: on the one hand, PI, as a binder, forms hydrogen bonds between PAA chains and Ti_3_C_2_T_*x*_ sheets, which enhances the interaction and the stress transfer between MXene sheets, further leading to an evenly dispersed tensile stress.^41^ On the other hand, the MXene/PI film exhibits a compact and well-ordered layer-by-layer structure due to the secondary orientation strategy (freeze-drying method and hot-pressing process), as proven by the analysis of XRD. The stacked MXene interlocking structure (Fig. 2(d)) is conducive to increasing the contact area and friction between sheets, leading to greater energy consumption during the relative slippage of the MXene sheets, and improves the break strength of the MXene/PI film further.^42^ Herein, the MXene/PI film shows good flexibility, as shown in the inset of Fig. 3(a).

### 2.5. Thermal management performance of MXene/PI films

The development of multifunction and miniaturization in modern electronics generates increasingly serious heat accumulation, which affects the efficiency and reliability.^22,38^ Therefore, efficient heat management is important for dissipating excessive heat. The thermal conductivity was evaluated by the laser flash method. Fig. 4(a and b) show the thermal conductivities and thermal diffusion coefficients of the MXene/PI film with different MXene weight ratios in the axial and radial directions, respectively. It can be seen that the thermal conductivities and thermal diffusion coefficients in both the axial and radial directions enhance with increasing weight fraction of MXene. Simultaneously, the MXene/PI film exhibits clearly anisotropic thermal characteristics. Apparently, corresponding to the in-plane efficient thermal diffusivity of 3.1 ± 0.22 mm^2^ s^−1^, the in-plane and through-plane thermal conductivities of the MXene/PI film reach 5.12 ± 0.37 W m^−1^ K^−1^ and 0.28 ± 0.006 W m^−1^ K^−1^, which exhibit an enhancement of approximately 5.7 times and 3 times compared to the PI film, respectively. Besides, Fig. 4(c) shows that the density of the MXene/PI film gradually increases with the increase of MXene content, and the specific heat capacity of the MXene/PI film is about 1.125 J g^−1^ K^−1^.^40^ In this work, we compared the thermal conductivity and mechanical tensile strength of different PI composite materials (Table S2†). In fact, the heat transfer of two-dimensional nanomaterials depends on the heat conduction performance of the materials and the formation of thermal networks strongly.^43^ Therefore, the reasons for the high thermal conductivity of the MXene/PI film may be as follows:^17,22–24,43^ (1) MXene has good dispersibility in the mixture solution and forms hydrogen bonds with PAA, which improves the interfacial compatibility between the filler and matrix. (2) PI can plug the gap between adjacent MXene sheets and enhance the interfacial adhesion. So, the interfacial thermal resistance can be reduced to the greatest extent due to a large contact area between MXene sheets in the in-plane direction. (3) The overlapped MXene sheets in the MXene/PI film can provide a high-speed channel for phonon conduction. Thus, the heat flow can be rapidly transmitted along the direction of the MXene layers. (4) The MXene/PI film exhibits a dense and well-ordered layer-by-layer structure due to the secondary orientation strategy, which results in more MXene sheets in the plane and further increases the conduction paths for heat transfer. To sum up, the MXene/PI film has a relatively high in-plane thermal conductivity. On the contrary, PI chains exist between MXene sheets along the direction of through-plane, resulting in slow heat transfer and low through-plane thermal conductivity.

To further characterize the heat dissipation performance, the temperature variation of PI and MXene/PI films at different times was recorded with an infrared camera. Herein, for accurate comparison, the center temperature is kept at the same level, and both surfaces of samples are coated with a thin layer of graphite paint to maintain the consistent surface emissivity and heat radiation.^32,44,45^ As shown in Fig. 4(d), the MXene/PI film exhibits a much faster cooling rate than the PI film under the same cooling conditions. After 30 seconds, the temperature of the MXene-40/PI film drops from 39.8 °C to 21 °C, while that of the PI film only decreased to 24.17 °C in the same situation. As shown in Fig. 4(e), all films are heated by a point heater under the same environment. With time goes up, the lateral thermal region increases and the central colors of all samples become brighter. Moreover, compared with PI, the composite films have a wider lateral area in the same time, indicating that the as-prepared MXene/PI film has better heat-spreading performance. As a result, the MXene/PI film has good heat transfer performance and the potential to be regarded as a promising candidate for thermal management.

### 2.6. Flame resistance of MXene/PI films

**Fig. 5:**
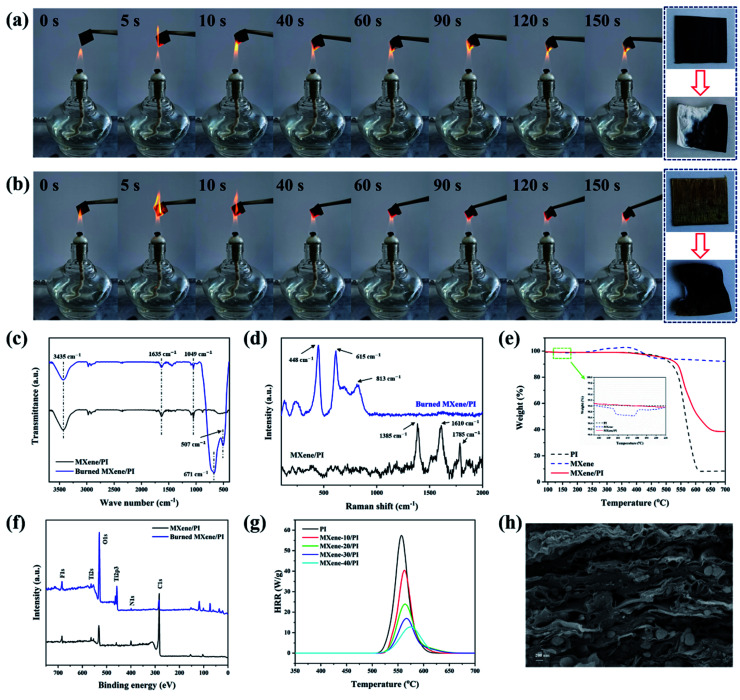
Flame resistance of MXene/PI films. Digital images of the (a) MXene-10/PI film and (b) PI film on a hot flame for different times. (c) FTIR spectra and (d) Raman spectra of burned and unburned MXene/PI. (e) TGA curves of PI, MXene and MXene/PI in an air atmosphere, respectively. (f) XPS survey spectra of the burned and unburned MXene/PI film. (g) HRR curves of all samples. (h) SEM image of the burned MXene/PI film.

The risk of fire is fatal for electronic equipment, especially for miniature and multifunctional devices, which are in danger of local overheating and short circuit.^7,46^ If the thermal management material has flame resistance, it can act as a barrier to prevent fire from spreading to a certain extent. Herein, the flame retardant tests of PI and MXene/PI films were carried out by burning them in the flame (800 °C) produced by an alcohol burner.^47,48^Fig. 5(a and b) show the combustion processes of MXene/PI and PI films under the consistent time and environment, respectively. And the whole demonstration process is shown in the Movies S1 and S2 of ESI.† It can be seen that both of them exhibit flame resistance for a long time, whereas the flame diffusion speed and burning area of the PI film are larger than those of the MXene/PI film. In addition, two new absorption peaks at 507 cm^−1^ and 671 cm^−1^ symbolizing the Ti–O stretching vibrations appeared in the burned MXene/PI film, suggesting the formation of TiO_2_ (Fig. 5(c)).^22^ However, in the Raman spectrum of Fig. 5(d), for the burned MXene/PI film, the amorphous and graphitic carbon peaks at 1610 cm^−1^ (G band) and 1385 cm^−1^ (D band) disappeared after burning, while the peaks belonging to TiO_2_ are obviously found at different positions (448 cm^−1^, 615 cm^−1^, and 813 cm^−1^).^49^ Besides, Fig. 5(e) shows that MXene has a weight loss of 1.3% at about 160 °C, indicating the formation of TiO_2_ according to the literature,^50,51^ and the final weight loss of the MXene/PI film is obviously less than that of the PI film. Fig. 5(f) shows the X-ray photoelectron spectroscopy (XPS) survey spectra of the burned and unburned MXene/PI film. The peaks of F 1s and N 1s can be found in the burned MXene/PI film, indicating that the removal of fluorinated and amino functional groups during the burning process was incomplete. Meanwhile, compared with the XPS spectrum of MXene/PI before burning, the peaks of O 1s and Ti 2p_3_ of burned MXene/PI become stronger, indicating the evolution of Ti_3_C_2_T_*x*_ MXene toward TiO_2_ during burning. And the peak of C 1s becomes weaker which may be mainly because the combustion products of PI were covered by the TiO_2_ layer during burning. Furthermore, the flame retardant behavior of PI and MXene/PI films was measured using a Micro-scale Combustion Calorimeter (MCC). As shown in Fig. 5(g) and Table S1,† PI and MXene/PI films show a sharp spike of the heat release rate (HRR) curve. With the addition of 40 wt% MXene, HRR decreased by 45 W g^−1^, the peak HRR decreased to 12.8 W g^−1^ and the corresponding maximum temperature increased by 16.7 °C, indicating that the flame retardancy of the composite film was improved. As shown in 5(h), it is clear that the layer structure of the MXene/PI film was kept well after combustion, and the formation of TiO_2_ can organize a physical protective layer to wrap the residue formed by PI carbonization. In addition, the results of SEM energy dispersive spectroscopy (EDS) of the MXene/PI film before and after burning (Fig. S2†) also demonstrate the protection effect of TiO_2_ during burning. There may be two reasons for these results:^22,38^ on the one hand, compared with the PI film, the MXene/PI film exhibits excellent thermal conductivity leading to rapid heat diffusion. On the other hand, the formation of TiO_2_ can organize a physical protective layer on the MXene–PI interface, which wraps the residue formed after PI carbonization, effectively preventing the combustion of the underlying materials and the propagation of the flame.

## 3. Conclusion

In summary, the MXene/PI film was fabricated using the secondary orientation strategy including the freeze-drying method and hot-pressing process. The resultant MXene/PI film exhibited a compact and well-ordered layer-by-layer structure, increasing the heat transfer path and improving the interfacial interaction in the MXene/PI film. Simultaneously, the composite film has good flexibility and flame retardancy owing to the synergistic effect between MXene sheets and PI chains. Therefore, those properties combined with high thermal and mechanical properties broaden their application fields and provide a novel and effective idea for preparing flexible, flame-retardant polymeric films with outstanding thermal conductivity and mechanical strength.

## 4. Materials and methods

### 4.1. Synthesis of the water-soluble PI precursor (PAA) solution

Poly(amic acid) (PAA) solution was synthesized using a polycondensation procedure in *N*,*N*-dimethylacetamide (DMAc) using an equivalent molar ratio of 4,4′-diaminodiphenyl ether (4,4′-ODA) and pyromellitic dianhydride (PMDA). The specific preparation process is detailed in the previous report of our group.^32^

### 4.2. Synthesis of MXene (Ti_3_C_2_T_*x*_) sheets

MXene (Ti_3_C_2_T_*x*_) sheets were synthesized by etching the aluminum (Al) layer from the Ti_3_AlC_2_ MAX phase according to the literature.^34^ Firstly, 1.6 g of lithium fluoride (LiF, 99%, Shanghai Aladdin Biochemical Co., Ltd) was dissolved in 20 mL of 9 M hydrochloric acid (HCl, 37%, Shanghai Aladdin Biochemical Co., Ltd) in a Teflon vessel. Then to etch its Al layer, 1 g of MAX powder (Ti_3_AlC_2_, 400 mesh, Shanghai Macklin Biochemical Co., Ltd) was slowly added into the LiF solution, and the reaction lasted for 30 h at 50 °C under stirring. Next, the resultant Ti_3_C_2_T_*x*_ was repeatedly washed with deionized (DI) water and centrifuged at 3500 rpm for 5 min until pH ≥ 6. Finally, the sediment was centrifuged at 1500 rpm for 30 min to obtain the self-delaminated Ti_3_C_2_T_*x*_ sheets and the homogeneous MXene (Ti_3_C_2_T_*x*_) sheet suspension (∼10 mg mL^−1^) was collected for further use.

### 4.3. Preparation of the MXene/PI film

The MXene/PI film was fabricated by means of two steps of the freeze-drying method and hot-pressing process, that is, the secondary orientation strategy. Fig. 1 shows the detailed preparation process of the MXene/PI film. Firstly, the PAA solution (50 mg mL^−1^) was mixed with the MXene (Ti_3_C_2_T_*x*_) suspension (10 mg mL^−1^) by stirring for 2 h. Then the resultant mixture was frozen and lyophilized for 96 h in a freeze-dryer (GZL-20, Beijing Songyuan Huaxing Science and Technology Development Co., Ltd.) to obtain the anisotropic MXene/PAA foam. The samples with different weight ratios of 10%, 20%, 30%, and 40% of MXene were denoted as MXene-*x*/PAA, where *x* was 10, 20, 30, and 40, respectively. Finally, the as-prepared MXene/PAA foam was subjected to hot-pressing treatment for 1 h in a (300 °C and 25 MPa) resistance vacuum hot-pressing furnace (ZYD-30–80, Jinzhou Aerospace Vacuum Equipment Co., Ltd) and the MXene/PI film was obtained.

### 4.4. Measurement and characterization

A field emission scanning electron microscope (SEM, model SU8000, Hitachi, Japan) was used to research the morphology and structure of the samples. X-ray diffraction (XRD) patterns were recorded with a Shimadzu XRD-7000s diffraction instrument (Cu Kα radiation, *λ* = 0.154 nm, 40 kV, 40 mA). Fourier transform infrared spectroscopy (FTIR) spectra were recorded using a Bruker Tensor-27 FTIR spectrometer. Raman spectroscopy (Renishaw InVia, England) was used to describe the production of Ti_3_C_2_T_*x*_. Thermal gravimetric analyses (TGA) were performed on a TG-DTA7300 thermal analyzer at a heating rate of 10 °C min^−1^ under an air atmosphere. The mechanical performance of the film was measured with a crosshead speed of 0.1 mm min^−1^ (Instron 5944, USA). Thermal conductivity (*K*) was calculated by *K* = *α* × *C*_p_ × *ρ*, where *C*_p_, *α* and *ρ* are the specific heat capacity, thermal diffusivity and density, respectively. The Netzsch LFA 467 light flash apparatus was used to measure the *C*_p_ and *a* at 27 °C. The infrared photos were recorded with an infrared camera (VarioCAM HD 880, German).

## Conflicts of interest

The authors declare no competing financial interest.

## Supplementary Material

NA-003-D1NA00415H-s001

NA-003-D1NA00415H-s002

NA-003-D1NA00415H-s003
