# Supplementary material for: Flame-retardant MXene/polyimide film with outstanding thermal and mechanical properties based on the secondary orientation strategy
Source: Nanoscale Adv. 2021 Aug 10;3(19):5683–93. doi: 10.1039/d1na00415h (PMC9419387; doi:10.1039/d1na00415h)
Supplement: NA-003-D1NA00415H-s003 [file NA-003-D1NA00415H-s003.pdf]

Electronic Supplementary Material (ESI) for Nanoscale Advances.
This journal is © The Royal Society of Chemistry 2021

Supplementary Information

Flame-retardant MXene/Polyimide Film with Outstanding Thermal and Mechanical Properties Based on the Secondary Orientation Strategy

Yue Zhu[I#], Qingyu Peng*[I II#], Haowen Zheng[I], Fuhua Xue[I], Pengyang Li[I], Zhonghai Xu[I], Xiaodong He[I II] and Xingbin Zhao[I]

[I] National Key Laboratory of Science and Technology on Advanced Composites in Special Environments, Center for Composite Materials and Structures, Harbin Institute of Technology, Harbin 150080, P. R. China

[II] Shenzhen STRONG Advanced Materials Research Institute Co., Ltd., Shenzhen 518000, P. R. China

*Corresponding authors. Email: pengqingyu@hit.edu.cn

[#] These authors contributed equally to this work.

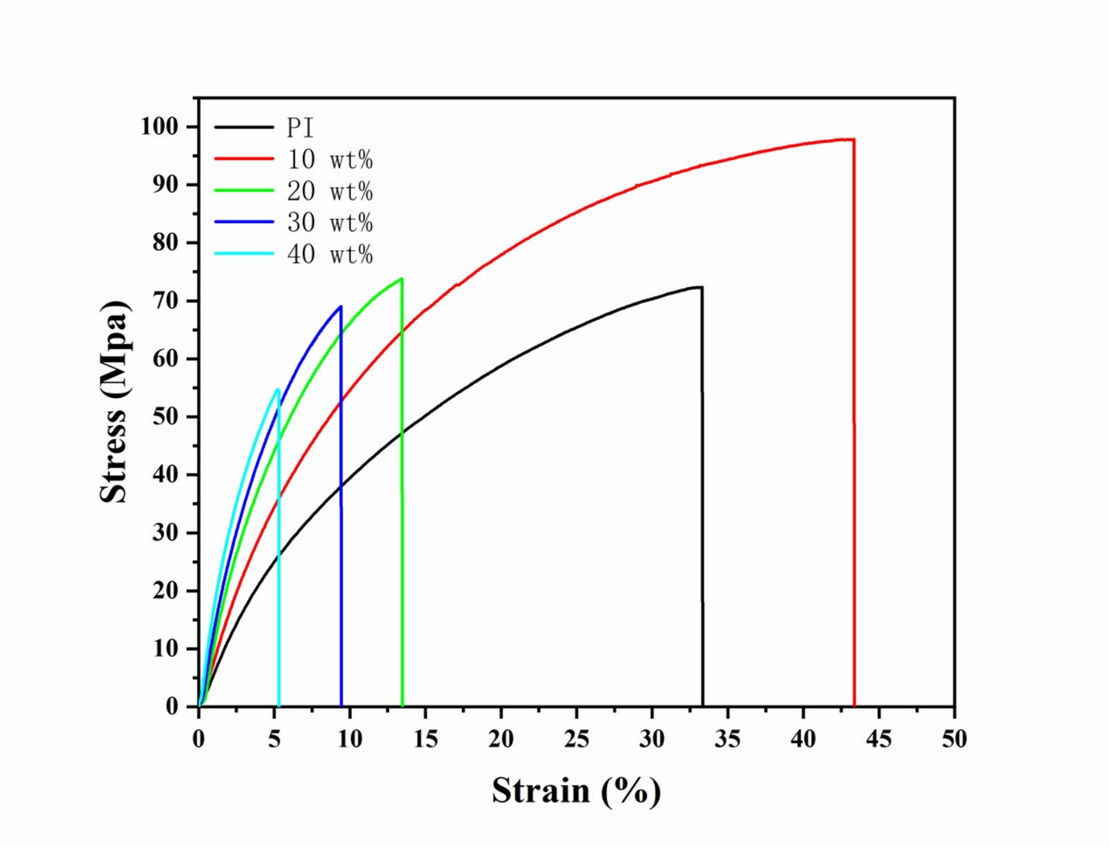

Fig. S1 Typical stress-strain curves of PI film and MXene/PI films.

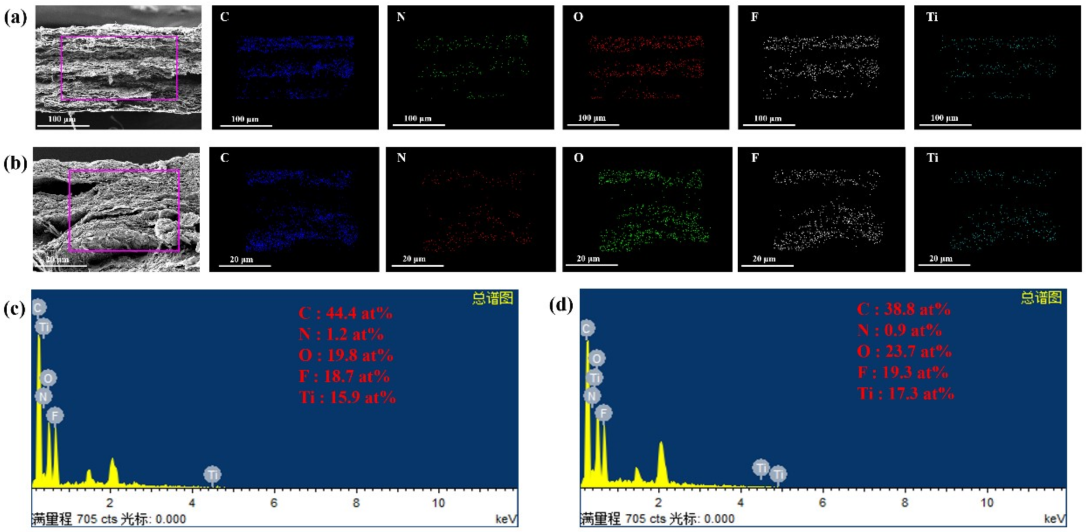

Fig. S2 SEM images of a) MXene/PI film, b) burned MXene/PI film and energy dispersive spectroscopy (EDS) mapping images of C, N, O, F, and Ti elements. EDS element spectrum and atomic percentage of c) MXene/PI and d) burned MXene/PI.

Table S1 Micro-scale Combustion Calorimeter (MCC) data of all samples.

Sample	HR Capacity (J/g-K)	Peak HRR (W/g)	Total HR (kJ/g)	Temperature (℃)
PI	75	47.6	0.9	556
MXene-10/PI	54	37	0.8	562.1
MXene-20/PI	28	22.7	0.8	563.1
MXene-30/PI	27	16.9	0.9	565.7
MXene-40/PI	26	12.8	0.7	572.7

Table S2 Comparison of properties of different PI composite materials.

Materials	Preparation method/condition	Thermal conductivity (W m^{-1} k^{-1})	Tensile strength (MPa)	Literature
BN/PI film	Casting method	1.16	~69.6	1
AlN/BN/PI film	Casting method	0.711	~120.1	2
NH_2-rGO/PI film	Knife Coating method	7.13	35.7	3
CNF/hBN/PI film	Dip Coating method	0.627	~70	4
G/PI film	Knife Coating method	0.2275	~127.5	5
PI/GO/BN film	Casting method	11.203	—	6
rGO/PI film	Casting method	2.78	—	7
BN/PI film	Casting method	2.58	—	8
PI/CNNS film	Solution Casting method	2.04	—	9
Hyperbranched PI film	Knife Coating method	—	124.1	10
PI/FGS film	Casting method	—	~122	11

ZnS-MPTMS/PI film	Knife Coating method	—	~87.7	12
FG/PI film	Casting method	—	65.76	13
**MXene/PI film**	**Secondary Orientation Strategy**	**~5.12**	**~102**	**Our work**

REFERENCES

[1] Zhang, G.-D.; Fan, L.; Bai, L.; He, M.-H.; Zhai, L.; Mo, S. Mesoscopic Simulation Assistant Design of Immiscible Polyimide/BN Blend Films with Enhanced Thermal Conductivity. *Chinese J. Polym. SCI* **2018**, *36* (12), 1394-1402.

[2] Liu, L.; Cao, C.; Ma, X.; Zhang, X.; Lv, T. Thermal conductivity of polyimide/AlN and polyimide/(AlN plus BN) composite films prepared by in-situ polymerization. *J. Macromol. SCI A* **2020**, *57* (5), 398-407.

[3] Ruan, K.; Guo, Y.; Lu, C.; Shi, X.; Ma, T.; Zhang, Y.; Kong, J.; Gu, J. Significant Reduction of Interfacial Thermal Resistance and Phonon Scattering in Graphene/Polyirnide Thermally Conductive Composite Films for Thermal Management. *Research* **2021,** *2021.*

[4] Haruki, M.; Tanaka, K. Controlling thermal conductivities and electrical insulation properties of carbon nanofiber/polyimide composites using surface coating techniques. *Polymer Composite* **2020**, *41* (8), 2990-2997.

[5] Wang, R.; Chen, M.; Li, Q.; Li, W.; Guo, Y.; Liu, L. Enhanced Mechanical and Thermal Properties of Polyimide Films Based on Functional Groups-Free Few-Layer Graphene. *J. Chem. Eng. JPN* **2019**, *52* (6), 570-578.

[6] He, X.; Wang, Y. Highly Thermally Conductive Polyimide Composite Films with Excellent Thermal and Electrical Insulating Properties. *Ind. Eng. Chem. Res.* **2020,** *59* (5), 1925-1933.

[7] Wei, S.; Yu, Q.; Fan, Z.; Liu, S.; Chi, Z.; Chen, X.; Zhang, Y.; Xu, J. Fabricating high thermal conductivity rGO/polyimide nanocomposite films via a freeze-drying approach. *RSC Adv.* **2018,** *8* (39), 22169-22176.

[8] Du, B. X.; Xiao, M. Effects of Thermally Conducting Particles on Resistance to Tracking Failure of Polyimide/BN Composites. *IEEE T. Dielect. E. L. In.* **2014**, *21* (4), 1565-1572.

[9] Wang, Y.; Zhang, X.; Ding, X.; Zhang, P.; Shu, M.; Zhang, Q.; Gong, Y.; Zheng, K.; Tian, X. Imidization-induced carbon nitride nanosheets orientation towards highly thermally conductive polyimide film with superior flexibility and electrical insulation. *Compos. B. Eng.* **2020,** *199*.

[10] Lei, X.; Qiao, M.; Tian, L.; Chen, Y.; Zhang, Q. Tunable Permittivity in High-Performance Hyperbranched Polyimide Films by Adjusting Backbone Rigidity (vol 120, pg 2548, 2016). *J. Phys. Chem. C* **2016,** *120* (40), 23304-23304.

[11] Nguyen Dang, L.; Hippi, U.; Korhonen, J. T.; Soininen, A. J.; Ruokolainen, J.; Johansson, L.-S.; Nam, J.-D.; Sinh, L. H.; Seppala, J. Enhanced mechanical and electrical properties of polyimide film by graphene sheets via in situ polymerization. *Polymer* **2011,** *52* (23), 5237-5242.

[12] Jeon, H.; Yoon, C.; Song, Y.-G.; Han, J.; Kwon, S.; Kim, S.; Chang, I.; Lee, K. Reducing the Coefficient of Thermal Expansion of Polyimide Films in Microelectronics Processing Using ZnS Particles at Low Concentrations. *ACS Appl. Nano Mater.* **2018**, *1* (3), 1076-1082.

[13] Zhang, P.; Zhang, K.; Chen, X.; Dou, S.; Zhao, J.; Li, Y. Mechanical, dielectric and thermal properties of polyimide films with sandwich structure. *Compos. Struct.* **2021**, 261.
